# Immunohistochemical analysis of S100-positive epidermal Langerhans cells in dermatofibroma^☆^^☆☆^

**DOI:** 10.1016/j.abd.2020.04.006

**Published:** 2020-07-12

**Authors:** Mahmoud Rezk Abdelwhaed Hussein

**Affiliations:** Department of Pathology, Assuit University Hospital, Assuit, Egypt

**Keywords:** Histiocytoma, benign fibrous, Skin neoplasms, S100 Proteins

## Abstract

Dermatofibroma is a dermal fibrohistiocytic neoplasm. The Langerhans cells are the immunocompetent cells of the epidermis, and they represent the first defense barrier of the immune system towards the environment. The objective was to immunohistologically compare the densities of S100-positive Langerhans cells in the healthy peritumoral epidermis against those in the epidermis overlying dermatofibroma (20 cases), using antibodies against the S100 molecule (the immunophenotypic hallmark of Langerhans cells). The control group (normal, healthy skin) included ten healthy age and sex-matched individuals who underwent skin biopsies for benign skin lesions. A significantly high density of Langerhans cells was observed both in the epidermis of the healthy skin (6.00 ± 0.29) and the peritumoral epidermis (6.44 ± 0.41) *vs.* those in the epidermis overlying the tumor (1.44 ± 0.33, p < 0.05). The quantitative deficit of Langerhans cells in the epidermis overlying dermatofibroma may be a possible factor in its development.

Langerhans cells (LCs) are the exclusive antigen-presenting cells of the normal human epidermis. They originate from two sources, the extra-embryonic yolk sac and fetal liver progenitors (monocytes). LCs represent about 3% of the cell population in the epidermis and are seen in histological sections stained with hematoxylin and eosin as “clear cells” in suprabasal layers. The expression of CD1a, langerin, and S100 is the immunophenotypic hallmark that distinguishes LCs from other dendritic cells.^1^

LCs have powerful functions in immune surveillance, and upon activation, they migrate from the epidermis to the lymph nodes. LCs can capture antigens, activate naive T-cells, and trigger a specific T-cell immune response with their clonal expansion and differentiation into effector and memory T-cells.^2^ Alterations of LCs occur in the peritumoral epidermis and the epidermis overlying the dermal tumors.^3^ Dermatofibroma is a common skin condition that usually affects the lower extremities in women. Some authorities regard dermatofibroma as a reactive process, but others consider that it is a benign mesenchymal tumor of fibroblasts, myofibroblasts, or even dermal dendrocytes.^4^ The current study compares the density of S100-positive LCs in the peritumoral epidermis and the epidermis overlying the neoplastic cells in dermatofibroma.

This retrospective study included formalin-fixed and paraffin-embedded skin specimens from 20 cases of dermatofibroma. The mean age of the patients was 49.40 ± 3.34 years. The specimens belonged to patients who had no history of immunological abnormalities. The control group (normal, healthy skin) included ten healthy age and sex-matched individuals who underwent skin biopsies for benign skin lesions as well as the healthy peritumoral skin (healthy skin adjacent to the tumors). The formalin-fixed and paraffin-embedded tissues were subjected to immunohistological analysis of LCs using antibodies against S100 antigen, utilizing a peroxidase/diaminobenzidine complex method following a previous study.^5^ The external controls included melanoma (for S100). The negative controls included sections running in parallel but with the omission of the primary antibodies (using PBS instead).^5^ The positive and negative controls yielded positive and negative results, respectively, indicating the validity of the results.

The immunoreactive LCs cells to S100 were counted using an Olympus light microscope, similarly to other groups.^6^ Imaging was done using a DFC450 digital camera. The epidermis was examined under the scanning magnifications, and the hot spots were determined. The S100-positive LCs per 1,000 cells were counted at 400× magnification. The immunoreactive LCs showing brown or red immunostaining and a visible nucleus, and dendrites were scored as positive. The results were reported as the mean and standard error of means. Student’s *t*-test was used, with p < 0.05 being considered statistically significant.

S100-positive LCs were seen in the spinous layer (cell bodies), with their dendrites extending into the suprabasal and cornified layers, both in the healthy epidermis (control group), peritumoral epidermis, and epidermis overlying the tumor (dermatofibroma). The cells were absent from the granular and cornified layers (Fig. 1). The densities of the S100-positive LCs were 6.00 ± 0.29 in the epidermis of the healthy skin and 6.44 ± 0.41 in the peritumoral epidermis. In dermatofibromas, the densities of S100-positive LCs varied between the peritumoral epidermis *vs.* the epidermis overlying the tumor. The mean density of these cells in the epidermis above the dermatofibroma was 1.44 ± 0.33. The variations in the densities of S100-positive LCs between the healthy skin (normal skin/control group and peritumoral epidermis) and tumoral epidermis were statistically significant (p < 0.000; Fig. 1).

**Figure 1:**
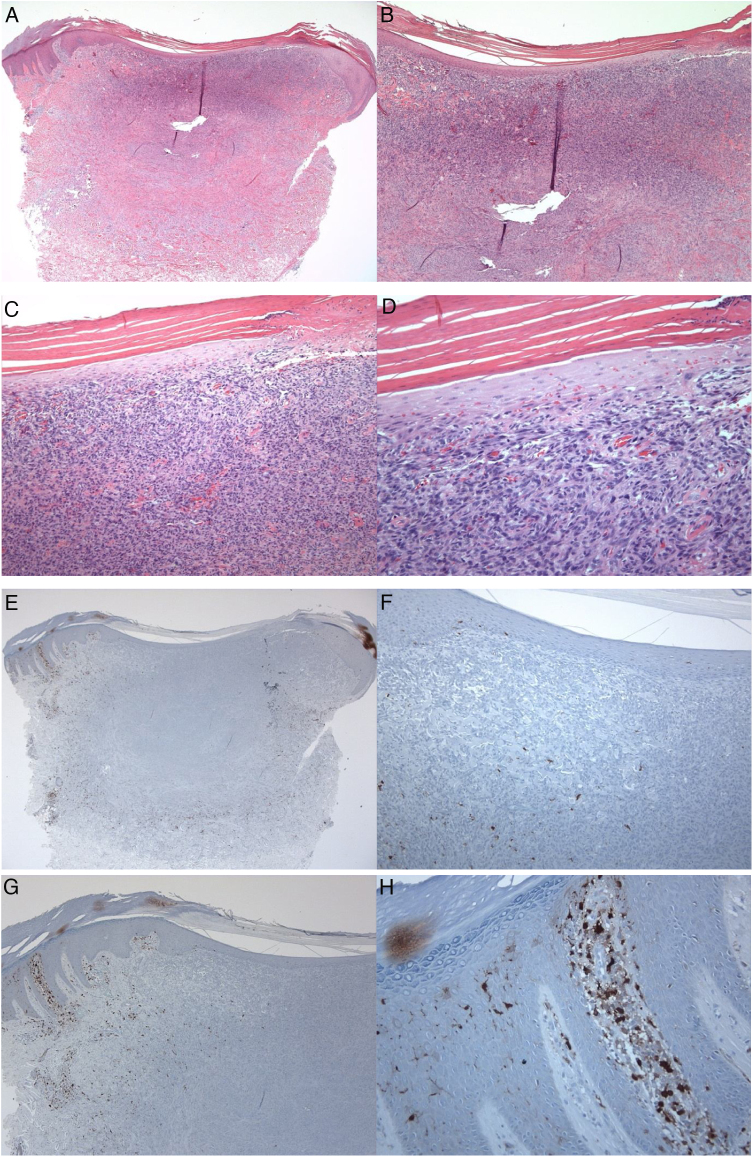
A 35-year-old male patient with a keratotic lesion of the left thigh. The clinical impression included dermatofibroma and acanthoma. A − D, Histologically, there is hyperparakeratotic epidermis overlying a poorly demarcated dermal growth composed of mitotically inactive interstitial spindle cells. Some areas of the tumor (upper portion) are densely cellular, while others (middle and lower portions) are sclerotic and hypocellular. The overlying epidermis is attenuated over the tumor *vs.*the hyperplastic peritumoral epidermis. Within the tumor, there is collagen trapping, thin-walled vessels, and hemorrhage (Hematoxylin & eosin). A x10; B x20; C x100; D x200. E–H, Peritumoral epidermis showing several S100-positive Langerhans cells located mainly in the spinous layer. Langerhans cells are almost absent from the epidermis overlying the tumor. Magnifications: A and E, ×20; B and F, ×40; C and G, ×100; D and H, ×400.

Next, LCs were examined and scored in the adjacent healthy peritumoral area of the epidermis and the epidermal area overlying the fibrohistiocytic neoplastic cells of the dermatofibroma. In this study, the numbers of LCs that expressed the S100 molecule were lower in the epidermis overlying the dermatofibroma, as compared with their numbers in the healthy peritumoral skin. These variations confirm the quantitative deficit of LCs as a possible factor in the development of dermatofibroma and are in agreement with the previous investigations in other skin tumors.^6^ Shevchuk et al. examined the expression values of LCs using antibodies against CD1a and langerin (phenotypic markers for LCs) using immunohistological formalin methods in healthy skin, actinic keratosis, basal cell carcinoma, and squamous cell carcinoma. The density of LCs was low in the basal cell carcinoma and squamous cell carcinoma as compared to the normal skin.^6^

The increased density of LCs in the peritumoral epidermis of dermatofibroma suggests strong immunologic response of this area to hinder the growth of the tumor, resulting in a more localized and restricted form of the dermal fibrohistiocytic proliferation. This upregulation of LCs may be due to alterations in the growth factors and cytokines,^7^ apoptosis, and the direct cytotoxic effects of the tumor cells. Several proteins are associated with the development, differentiation, and maintenance of LCs, such as transforming growth factor-beta (TGFβ), IL-34, BMP-7, integrin (ITG) avb6, and ITGavb8.^7^, ^8^ This author proposes that the increased density of LCs in the peritumoral epidermis of dermatofibroma is due to upregulation of these molecules. Activins A (TGFβ family members) can induce the differentiation of human monocytes into LCs and therefore are important for populating the epidermis with LCs.^9^ Itching (mechanical trauma) and superimposed inflammatory changes are common events associated with dermatofibroma (irritated or inflamed dermatofibroma). It is possible that this mechanical trauma and the associated inflammatory response represent the underlying mechanisms for the loss of LCs in the epidermis overlying the tumor. In support, LCs leave the epidermis LCs in response to inflammatory stimuli or gentle tape stripping.^10^ It is still possible that the neoplastic cells of dermatofibroma exert direct cytotoxic effects on LCs, and the loss of these cells may be a reflection of these effects.

To conclude, the current study revealed a reduction in the numbers of epidermal S100-positive LCs overlying the tumor areas as compared to those in the peritumoral epidermis. It is worthwhile to examine the alterations of the molecules contributing to quantitative deficit of LCs in the epidermis overlying dermatofibroma, such as TGFβ, IL-34, BMP-7, ITGavb6, and ITGavb8.^7^, ^8^

## Financial support

None declared.

## Authors’ contributions

Mahmoud Hussein: Statistical analysis; approval of the final version of the manuscript; conception and planning of the study; drafting and editing of the manuscript; collection, analysis, and interpretation of data; participation in study design; intellectual participation in the propaedeutic and/or therapeutic conduct of the studied cases; critical review of the literature; critical review of the manuscript.

## Conflicts of interest

None declared.
